# Chronic Atrial Fibrillation Ablation with Harmonic Scalpel during Mitral Valve Surgery

**DOI:** 10.21470/1678-9741-2016-0015

**Published:** 2017

**Authors:** Alexandre Visconti Brick, Domingo M. Braile

**Affiliations:** 1 Surgery Department of Faculdade de Medicina da Universidade de Brasília (FM-UnB), Brasília, DF, Brazil.; 2 Faculdade de Medicina de São José do Rio Preto (FAMERP), São José do Rio Preto, SP, Brazil and Universidade de Campinas (UNICAMP), Campinas, SP, Brazil.

**Keywords:** Atrial fibrillation, Arrhythmias, cardiac, Ablation techniques, High-intensity focused ultrasound ablation, Cardiovascular surgical procedures, Mitral Valve Disease

## Abstract

**Objective:**

To evaluate surgical treatment of chronic atrial fibrillation with ultrasound in patients with mitral valve disease, considering preoperative clinical characteristics of patients undergoing surgical procedure and follow-up in the immediate postoperative period, in hospital and up to 60 months after discharge.

**Methods:**

We studied 100 patients with chronic atrial fibrillation and mitral valve disease who underwent surgical treatment using ultrasound ablation. Patient data were reviewed by consulting the control reports, including signs and symptoms, underlying disease, functional class, hospital stay, surgical procedure time, ablation time, immediate complications, and complications at discharged and up to 60 months later. Actuarial curve (Kaplan-Meier) was used for the study of permanence without recurrence after 12, 24, 36, 48 and 60 months.

**Results:**

86% of the patients had rheumatic mitral valve disease, 14% had degeneration of the mitral valve, 40% had mitral regurgitation, and 36% had mitral stenosis. Main symptoms included palpitations related to tachycardia by chronic atrial fibrillation (70%), congestive heart failure (70%), and previous episodes of acute pulmonary edema (27%). Early results showed that 94% of the patients undergoing ultrasound ablation reversed the rate of chronic atrial fibrillation, 86% being in sinus rhythm and 8% in atrioventricular block. At hospital discharge, maintenance of sinus rhythm was observed in 86% of patients and there was recurrence of chronic atrial fibrillation in 8% of patients. At follow-up after 60 months, 83.8% of patients maintained the sinus rhythm.

**Conclusion:**

Surgical treatment of chronic atrial fibrillation with ultrasound concomitant with mitral valve surgery is feasible and satisfactory, with maintenance of sinus rhythm in most patients (83.8%) after 60 months of follow-up.

**Table:** 

Abbreviations, acronyms & symbols	
AF CAF CPB ISMICS NYHA US	= Atrial fibrillation = Chronic atrial fibrillation = Cardiopulmonary bypass = International Society of Minimally Invasive Cardiothoracic Surgery = New York Heart Association = Ultrasound

## INTRODUCTION

Atrial fibrillation (AF) is the most common and the most complex supraventricular arrhythmia, characterized by loss of atrial contraction, and its diagnosis is easily accomplished by clinical manifestation, cardiac auscultation, and electrocardiogram. The prevalence of AF is estimated at 0.4% in the general population and it increases proportionately with age, occurring in less than 1% of those below 60 years old and more than 6% in patients over 80 years old^[1]^. Regarding gender, its incidence is higher in men^[2]^. The occurrence of AF may be associated with other cardiopulmonary diseases, such as valvular disease, especially with the involvement of the mitral valve^[3]^. AF is termed chronic when it persists for more than a year or continues after the first episode.

The non-pharmacological treatment of AF includes techniques such as intraoperative cryoablation of the His bundle and the atrioventricular junction^[4]^; electrical cardioversion, with a high recurrence rate; left atrial isolation^[5]^; catheter ablation of the His bundle and permanent pacemaker implantation; catheter ablation of the atrioventricular junction and permanent pacemaker implantation; and “Corridor operation”.

Electrophysiological mapping studies with computerized electrode system were performed by Cox et al.^[6]^, who developed the surgical technique known as maze procedure. It consists of making incisions and sutures on the atrial wall, allowing the spread of electrical stimulation in the atria within a maze, and setting atrial rate.

The applicability of surgical techniques is inversely proportional to their complexity. In order to reduce the complexity of the “Cox operation”^[7]^, several technical changes were introduced, such as modifications in the locations of atrial incisions; reduction of section lines and sutures on the atrial wall, known as “Mini Cox”^[8]^; and a unilateral procedure, only in the left atrium, called “Cox at Left”.

In Brazil, Jatene et al.^[9]^ evaluated the results of late evolution of the Cox maze procedure for chronic atrial fibrillation (CAF) in patients with mitral valve disease. In addition, it has been demonstrated that, in patients with CAF and valvular disease, a combination of valve repair and completion of the Maze technique devised by Cox allows for the return to sinus rhythm^[10]^. Although of proven efficiency, the “maze technique”, as it is generally performed, uses dissection and opening of the atrial wall, followed by suture, which increases cardiopulmonary bypass (CPB) time and the chance of complications in the postoperative period.

The use of catheter ablation for the treatment of supraventricular arrhythmias stimulated the use of energy sources to cause linear ablative lesions, with either endocardial or epicardial application or both, replacing the section and suture of the atrial wall^[11,12]^. New energy sources capable of causing permanent blockage lines have been used^[13]^ such as radiofrequency^[14-18]^, microwave^[19]^ (90=17), and ultrasound (US)^[20,21]^.

This author, considering the experience with an US scalpel in the surgical treatment of refractory ventricular tachycardia^[22]^, devised a new approach, applying US and using the scalpel (UltraCision^®^) to form injury lines that would determine the partitioning of the left and right atria, with less surgical and CPB time and consequent reduction in postoperative complications.

Several aspects justified this research, namely: difficulties in establishing drug treatment or other drugs indicated to eliminate CAF; complications caused by CAF, especially thromboembolism with increasing morbidity and mortality; presence of previous heart disease, most often injury of the mitral valve; advances in the study of the electrophysiological mechanisms of arrhythmia; description of the focal origin mechanism of AF in the pulmonary veins, as well as foci located in the vena cava; and demonstration that the ablations performed with US systems, through both endocardial and epicardial, are likely to cause transmural lesion. Thus, the aim of this study was to evaluate the surgical treatment of CAF with US in patients with mitral valve disease, considering preoperative clinical characteristics of patients undergoing surgical treatment for CAF and follow-up in the immediate postoperative period, at hospital discharge, and late postoperative of up to 60 months.

## METHODS

We retrospectively and consecutively studied 100 patients with CAF and mitral valve disease with indication for surgical treatment, regardless of race, from various regions of the country, aged between 18 and 70 years (43.56±4.94 years), and with 63 (63%) being female and 37 (37%), male. The patients underwent surgery by the author in the period between 1999 and 2004 and were surgically treated by ablation with US. All patients were treated at Hospital das Forças Armadas and Brasília Hospital, located in Brasilia/DF, Brazil.

Exclusion criteria were: pregnant women; children under 16 years old; AF patients who were not considered chronic, with or without valvular heart disease; people with coronary, congenital and vascular diseases; transplant or indication for heart transplantation; patients undergoing two reoperations of the mitral valve; and those with other isolated or associated valve diseases (except the tricuspid valve disease). This study was approved by the Research Ethics Committee of São José do Rio Preto Medical School (FAMERP) (Protocol No. CAAE 37374414.9.0000.5415).

Patient data were reviewed prospectively by consulting control records, including demographic variables (gender and age). Cardiac variables included clinical presentation (signs and symptoms), underlying disease, functional class, length of hospital stay (days), surgical procedure time (minutes), ablation time (minutes), intraoperative and postoperative complications (immediate, at hospital discharge and late postoperative, up to 60 months).

All patients had indication for mitral valve repair or replacement, in some cases for tricuspid valve repair and correction of CAF. US was used to perform the intraoperative ablation lines, in accordance with Brick et al.^[23]^, using harmonic scalpel UltraCision®, trademark of Ethicon Endo Surgery, Division of Johnson & Johnson Professional Products (São Paulo, SP, Brazil).

After anoxic arrest, longitudinal left atriotomy was performed by clamping of the aorta. The ablation lines were carried out in inverted U-shape, surrounding the four pulmonary veins, by starting and finishing at the fibrous annulus of the heart toward the mitral valve. In patients with thrombi in the atrium and left atrium, thrombectomy was performed before ablation. Upon completion of the ablation lines, routine procedures were done for treatment of mitral disease with replacement or repair.

In the right atrium, after longitudinal opening with a beating heart, linear ablations were performed in the interatrial septum: 1- from around the superior vena cava to around the inferior vena cava; 2- from the inferior vena cava to the bottom edge of the tricuspid valve annulus, passing close to the coronary sinus ostium, and 3- from the superior vena cava to the upper portion of the tricuspid annulus, taking care of the atrioventricular node.

A daily dose of 200 mg of amiodarone was maintained for six months for atrial remodeling and stabilization.

Data were analyzed using descriptive statistics (mean, standard deviation, median, minimum and maximum). The actuarial curve (Kaplan-Meier) for the study of permanence without recurrence after 12, 24, 36, 48 and 60 months in patients with CAF with confidence interval of 95% was used. The actuarial curve (Kaplan-Meier) was performed using the statistical program Statistical Calculations for Windows v.1.8.

## RESULTS

**Fig. 1:**
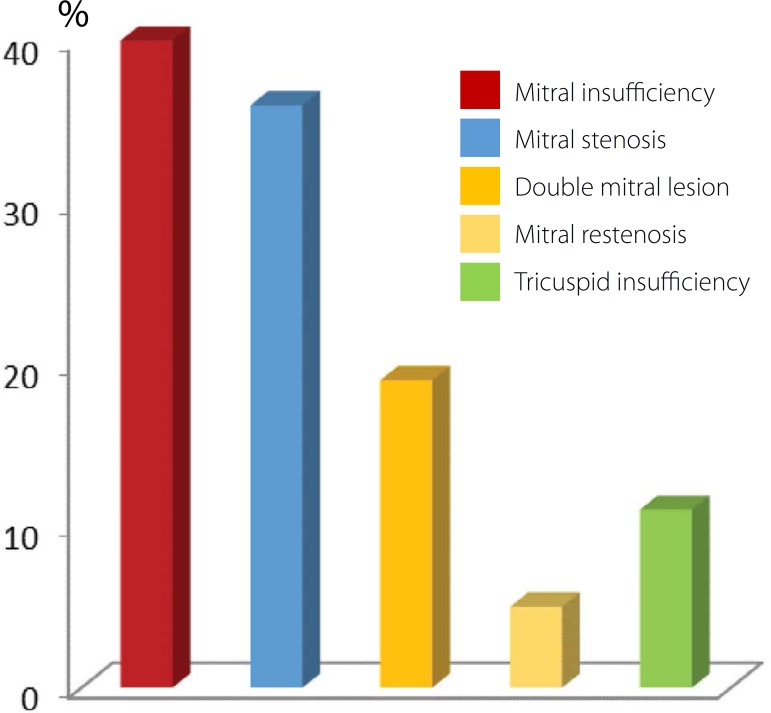
Percentage distribution of valvular disease in patients with chronic atrial fibrillation.

Of the patients studied, 86% had rheumatic mitral valve disease and 14% presented degeneration of the mitral valve. The analysis of valvular disease showed that 40% of the patients had mitral regurgitation, 36% had mitral stenosis, 19% had double mitral lesion, and 5% had mitral restenosis (Figure 1). In addition to mitral valve disease, 11% of the patients had associated tricuspid valve regurgitation, which was also corrected with valvuloplasty during the procedure.

**Fig. 2:**
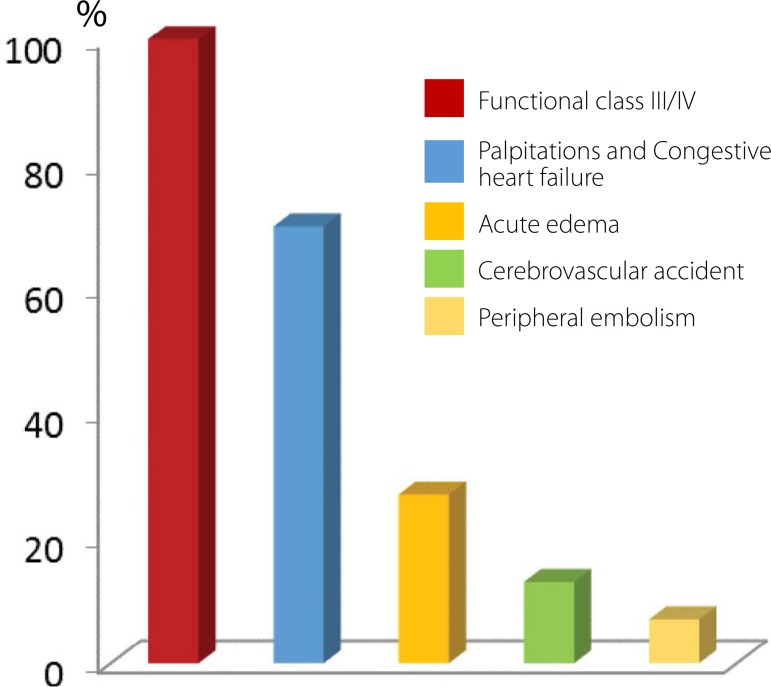
Percentage distribution of symptoms and functional class in patients with chronic atrial fibrillation.

Main symptoms included palpitations related to tachycardia by CAF (70%), congestive heart failure (70%), previous episodes of acute pulmonary edema (27%), stroke due to thromboembolism (13%), and peripheral embolism (7%); these patients required embolectomy with Fogarty catheter (Figure 2). The functional class of patients, according to New York Heart Association (NYHA), was III/IV and the average size of the left atrium measured on echocardiography was approximately 60 mm.

**Fig. 3:**
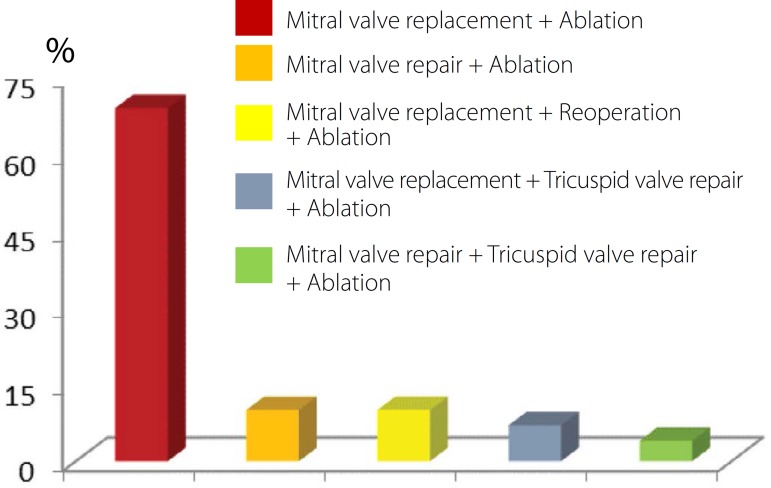
Procedures performed concurrently with the CAF ablation.

**Fig. 4:**
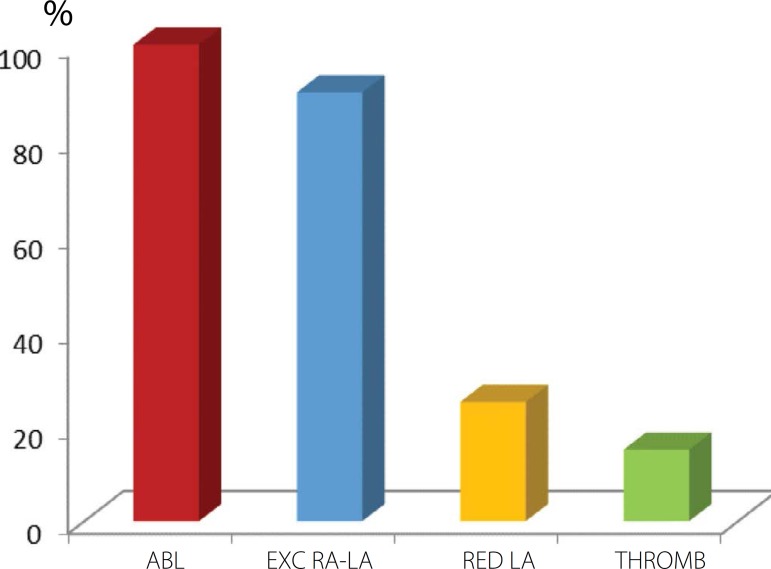
Supplementary procedures to surgery of US ablation. ABL=ablation; EXC RA-LA=exclusion of the right and left atria; RED LA=reduction of the left atrium; Thromb=thrombectomy

Operations performed concurrently with the ablation included mitral valve replacement for bovine pericardial bioprosthesis of Braile Biomédica® (São José do Rio Preto, SP, Brazil) (69%), mitral valve repair (10%), reoperation with valve replacement (10%), and associated with tricuspid valve (11%) (Figure 3). In addition to ablation with US, in all patients, additional procedures to surgery included: exclusion of the right and left atria (90%), reduction in the size of the left atrium through resection and/or plication of the left atrial wall (25%), and thrombectomy (15%) (Figure 4).

**Fig. 5:**
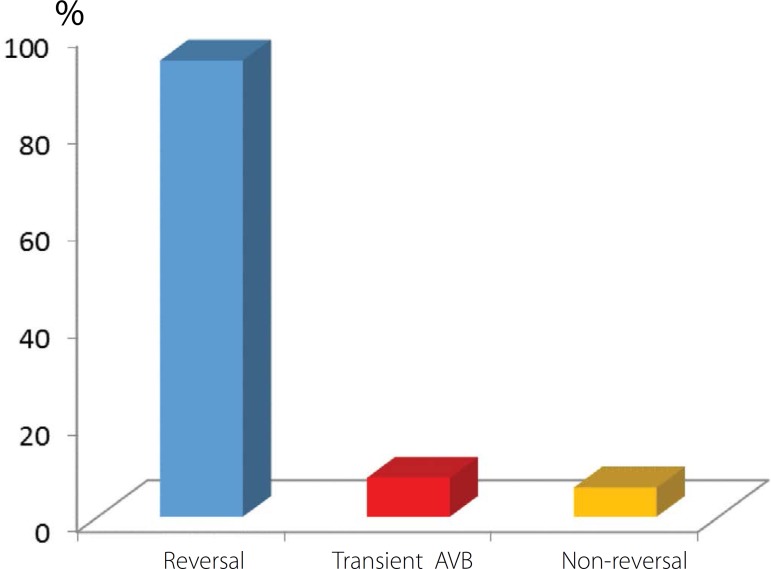
Results obtained in the immediate postoperative period for patients undergoing US ablation. AVB=atrioventricular block

Early results showed that 94% of patients undergoing US ablation reversed the rate of CAF, 86% being in sinus rhythm and 8% in atrioventricular block, which was transient. Among 6% of the patients, there was no success, there were 4 reoperations, and left atrium with severe fibrosis caused by rheumatic fever and/or calcified (Figure 5). At the end of the procedure and at the end of CPB, 96% of patients maintained adequate cardiac output, even when CAF was not reversed. There was no reoperation for bleeding in the immediate postoperative period. In one patient, there was injury of the left atrium wall by US scalpel, corrected by suturing the left atrium. Median operation time was 142 minutes with anoxic arrest of 45 minutes. The time to perform the ablation lines were 12.5 and 14 minutes for the right and left atria, respectively (Table 1).

**Table 1:** Results on the median time (in minutes) of the surgical treatment of patients with chronic atrial fibrillation and mitral valve disease.

Time of Surgery	CPB time	Time of arrest	Ablation Time	
			RA	LA
142	72.5	45	12.5	14
120-210	45-100	20-70	10-13	11-14

CPB=cardiopulmonary bypass; RA=right atrium; LA=left atrium

Analyzing the results at hospital discharge, we observed maintenance of sinus rhythm in 86% of patients and recurrence of CAF in 8%. Hospital stay ranged from 5 to 12 days, with an average of 6.6 days. Four hospital deaths were recorded, two patients who underwent reoperation for calcified mitral restenosis had respiratory failure in the postoperative period, and two patients who had preoperative functional class IV and an episode of acute edema evolved with low output syndrome and unmanageable heart failure. These 4 patients had AF in the immediate postoperative period. One patient had sternal dehiscence.

**Fig. 6:**
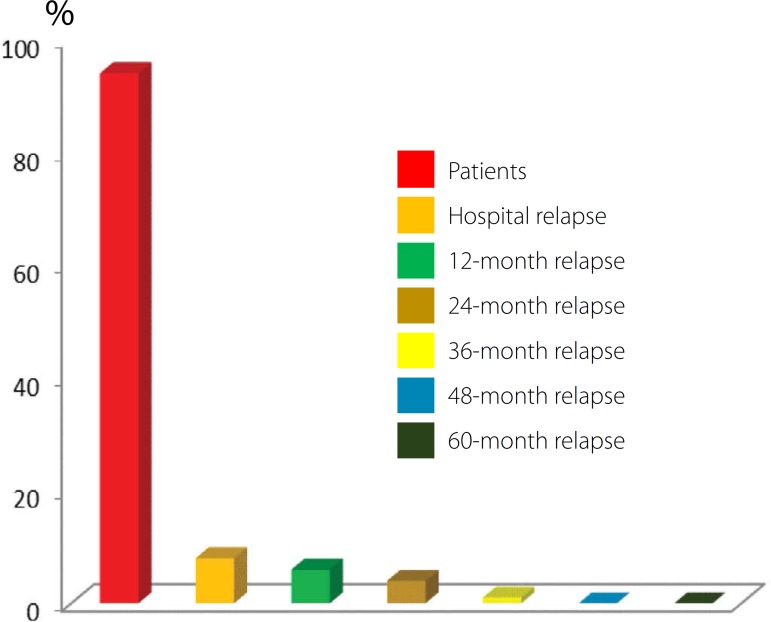
Results obtained postoperatively with respect to relapse of chronic atrial fibrillation in patients undergoing US ablation.

**Fig. 7:**
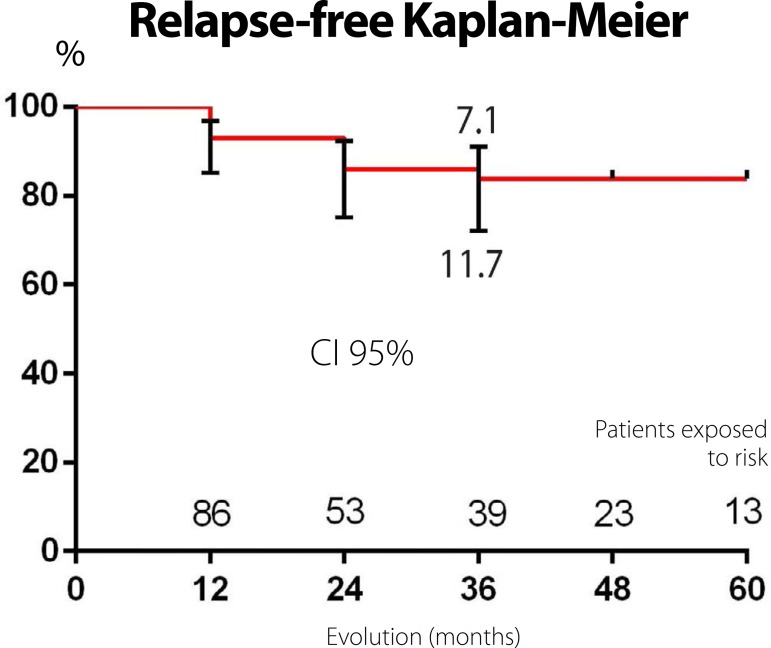
Actuarial curve (Kaplan-Meier) for probability of remaining without recurrence up to 60 months of follow-up in 86 patients with chronic atrial fibrillation who underwent surgery with US.

After hospital discharge, patients were followed-up in outpatient clinics with conduction of CPB, 24-hour Holter, and echocardiogram, initially and at 15, 30, and 60 days postoperatively. Clinical follow-up of late postoperative period was performed at 12, 24, 36, 48 and 60 months after hospital discharge. Figure 6 shows the results of the relapse in the postoperative period. Follow-up time ranged from 12 to 60 months. At follow-up after 60 months, 83.8% of patients maintained relapse-free sinus rhythm (Figure 7).

## DISCUSSION

The results of this research show that the surgical treatment of chronic AF with US in patients with mitral valve disease is feasible. In most patients with CAF, rheumatic mitral valve disease, mitral regurgitation, palpitation related to tachycardia, and congestive heart failure are more frequent. The low rate of intraoperative and postoperative complications shows that surgical ablation of CAF with US concomitant with valve surgery is safe and effective. Maintenance of sinus rhythm rate after 60 months of follow-up for these patients shows the benefits of surgical ablation with US in the treatment of this arrhythmia.

Regarding functional class, all patients showed considerable improvement, staying mostly in class I or II, which indicates that maintenance of the rhythm with contraction of the atria positively influenced the results. In this study, although they are important in the evolution of patients and can influence the final results, variables such as size of the left atrium, left ventricular ejection fraction and functional class of CHF (NYHA) after surgery were not analyzed because what mattered was arrhythmia with maintenance or not of sinus rhythm.

Faced with poor results of clinical treatment and increased incidence of CAF, the pioneering study of Cox et al.^[6]^ demonstrated the ability to surgically treat AF. Kong et al.^[24]^ compared, through meta-analysis, the effectiveness of the Maze procedure with concomitant cardiac surgery *versus* drug therapy for treatment of patients with valvular AF. They concluded that surgical treatment is associated with reduced AF after 1 year, with no significant increase in the average length of hospital stay, postoperative complications or mortality from all causes.

Comparing the results of several studies in the literature on surgical treatment of CAF is difficult due to the large number of variables analyzed and the different classifications and treatment techniques. In a recent literature review, Brick & Braile^[12]^ identified 72 studies on evolution and improvement of surgery of arrhythmias. Analyzing studies with immediate results, the percentage of return to sinus rhythm ranged from 73% to 96%, while those of long-term results (12 months on) ranged from 62% to 97.7%. Both showed consequent clinical improvement of the patients who underwent ablation, regardless of the energy source used.

In this research, there was a careful attempt to standardize the sample, eliminating patients with other diseases developing CAF, such as congenital and coronary artery disease. Only patients with mitral and tricuspid valves were concurrently treated because performing ablation also in the right atria is important to eliminate possible sources of atrial flutter.

In this study, it was found that 75% of patients in a follow-up of 60 months were in sinus rhythm after mitral valve surgery associated with ablation of the CAF using US as energy source. This result is consistent with the literature^[12]^. Meta-analysis of results of 62 studies on surgical ablation in patients with AF undergoing mitral valve surgery show a significantly higher rate of sinus rhythm in these patients, with no increase in mortality rate^[16]^.

The ideal energy source would be fast, reliable, producing transmural lesions that would not damage the surrounding tissues, and capable of being used with CPB in minimally invasive endocardial and epicardial application. The first patients who underwent surgery by the author for correction of CAF with unipolar radiofrequency catheter were not included in this analysis. The development of bipolar ablation devices with irrigated catheter contributed to the technical improvement of the procedure with consequent shorter surgery and satisfactory operative results, showing a 96% rate of reversion to sinus rhythm^[6]^. It was followed by the emergence of the use of the US scalpel, which seemed more appropriate since it facilitated the procedure, with better results and less surgery time.

Benussi et al.^[25]^ described radiofrequency application in patients with mitral valve disease. The procedure, similar to that performed by the author, also surrounded the right and left pulmonary veins, with exclusion of the left atrial appendage. After 36 months of development, 77% of the patients (n=132) were free of AF. In our series, after 60 months, sinus rhythm maintenance percentage, at 83.8%, was higher than that obtained by Benussi et al.^[25]^.

For this author^[6]^, the use of US with harmonic scalpel (UltraScision^®^) began after experiencing initial difficulty with a proper catheter to perform surgical radiofrequency ablation and using the US scalpel in surgery for treatment of refractory ventricular tachycardia in Chagas disease patients with left ventricular aneurysm. Currently, several devices have been developed using US for both endo- and epicardial application, facilitating the procedure and making surgery less invasive and safer.

The success rate of CAF reversal of this study is compared to that of other authors^[15,20,26]^, using various energy sources and techniques, such as radiofrequency, cryoablation, and US, and performing the atrial ablation lines in the same way.

In this series, analyzing immediate and hospital outcomes, maintenance of sinus rhythm was observed in 94% and 86% of patients, respectively. Lins et al.^[20]^, when comparing patients undergoing US ablation to those who did not, found that 80% of the patients treated with US were free of AF whereas, in the other group, that occurred to only 30% of the patients. The results observed in this study are similar to those obtained in the study presented herein, showing that US ablation can be applied in patients with surgical indication for mitral valve disease repair.

In this research, the right atrium approach (biatrial) was performed in all patients because it was considered important to treat and prevent atrial flutter, which is related to the vena cava and right atrium. When comparing results obtained in patients with AF associated with mitral valve disease who underwent left or biatrial atrial ablation, Kim et al.^[27]^ found that biatrial ablation was more effective in restoring and maintaining sinus rhythm without increasing the risk of postoperative complications.

In this series, the operative results were also safe, with complications related to the ablation procedure (lesion in the left atrium) in only one patient and immediate results of reversion to sinus rhythm of 94% and 83.8% after 60 months. The reduction of recurrence rates to maintain sinus rhythm can be observed over time.

The operative times in this study were satisfactory compared with other techniques for the surgical treatment of CAF. Median time of operation was 142 minutes with anoxic arrest of 45 minutes. The time to perform the ablation lines was 12.5 minutes in the right atrium and 14 minutes in the left atrium.

The complications in the postoperative period, such as low cardiac output syndrome and respiratory failure, were not related to the non-reversal of arrhythmia; however, due to complications after surgery, those patients did not maintain sinus rhythm. In this study, mortality rate was 4%, unrelated to arrhythmia.

AF has been neglected for being considered benign. However, it is associated with many potentially lethal complications, with high morbidity and mortality rates. Some questions should be asked for the treatment of AF, namely: Is it reversible? What is the arrhythmia time? Are there related symptoms? Is there a structural disease base? In the case of mitral valve disease with surgical indication, what is the age and clinical condition of the patient? And, finally, what is the best treatment option and the impact on morbidity and mortality?

In addition to the endocardial application, US and other devices can be used in off-pump surgery, through epicardial application in patients with isolated fibrillation. The aim of the Consensus of the International Society for Minimally Invasive Cardiothoracic Surgery (ISMICS)^[28]^ was to determine whether surgical ablation of AF during associated cardiac procedures improves postoperative clinical outcomes. The group involved in the study analyzed the best available evidence, with systematic data review, including randomized and nonrandomized controlled studies, always in descending order of importance. A systematic review with meta-analysis identified 10 randomized trials (650 patients) and 23 non-randomized (3997 patients) trials, mostly published in English and performed in the United States.

The authors of the consensus study defined the following recommendation: in patients with persistent and permanent AF, surgical ablation is recommended to increase the incidence of sinus rhythm in the short- and long-term (Class 1, Level A); reduce the risk of stroke and thromboembolic events (Class 2a, level B); increase exercise tolerance and improve ventricular function (Class 2a, level A); and increase survival (Class 2a, level B)^[28]^.

The use of the US technique for treatment of CAF with mitral valve disease is easy to perform, with a low complication rate and minimum surgical and CPB time. It can be reproduced by several cardiac surgery centers, requiring only the US scalpel, which exists in most hospitals that perform laparoscopic surgery. From the classic maze surgery (Cox Maze), changes in AF surgery have been occurring using alternative energy sources. The results of the surgical ablation of the CAF in patients with mitral valve disease depend on the energy source used, the lesion produced being transmural and applied in the two atria, and, with current technology, being minimally invasive.

## CONCLUSION

Patients with rheumatic mitral valve disease often have mitral valve failure and mitral stenosis, palpitations related to tachycardia by CAF, and congestive heart failure. Surgical treatment of CAF with US concomitant with mitral valve surgery is feasible and satisfactory, with maintenance of sinus rhythm in most patients (83.8%) after 60 months of follow-up.

**Table:** 

Authors’ roles & responsibilities	
AVB	Study design; writing of the manuscript or critical review of its content; final approval of the manuscript
DMB	Study design; writing of the manuscript or critical review of its content; final approval of the manuscript
